# Use acupuncture to treat functional constipation: study protocol for a randomized controlled trial

**DOI:** 10.1186/1745-6215-13-104

**Published:** 2012-07-03

**Authors:** Ying Li, Hui Zheng, Fang Zeng, Si-yuan Zhou, Feng Zhong, Hua-bing Zheng, Min Chen, Xiang-hong Jing, Yu-ying Cai, Bao-hui Jia, Bing Zhu, Zhi-shun Liu

**Affiliations:** 1 Chengdu University of Traditional Chinese Medicine, Chengdu, Sichuan, China; 2 First Affiliated Hospital of Chengdu University of Traditional Chinese Medicine, Chengdu, Sichuan, China; 3 Guang’anMen Hospital, China Academy of Chinese Medical Sciences, Beijing, 100053, China; 4 Institute of Acupuncture and Moxibustion, China Academy of Chinese Medical Sciences, Beijing, 100700, China

**Keywords:** Acupuncture, Functional constipation, Study protocol

## Abstract

**Background:**

Whether acupuncture is effective for patients with functional constipation is still unclear. Therefore, we report the protocol of a randomized controlled trial of using acupuncture to treat functional constipation.

**Design:**

A randomized, controlled, four-arm design, large-scale trial is currently undergoing in China. Seven hundred participants are randomly assigned to three acupuncture treatment groups and Mosapride Citrate control group in a 1:1:1:1 ratio. Participants in acupuncture groups receive 16 sessions of acupuncture treatment, and are followed up for a period of 9 weeks after randomization. The acupuncture groups are: (1) Back-Shu and Front-Mu acupoints of Large Intestine meridians (Shu-Mu points group); (2) He-Sea and Lower He-Sea acupoints of Large Intestine meridians (He points group); (3) Combining used Back-Shu, Front-Mu, He-Sea, and Lower He-Sea acupoints of Large Intestine meridians (Shu-Mu-He points group). The control group is Mosapride Citrate group. The primary outcome is frequency of defecation per week at the fourth week after randomization. The secondary outcomes include Bristol stool scale, the extent of difficulty during defecating, MOS 36-item Short Form health survey (SF-36), Self-Rating Anxiety Scale (SAS), and Self-rating Depression Scale (SDS). The first two of second outcomes are measured 1 week before randomization and 2, 4, and 8 weeks after randomization. Other second outcomes are measured 1 week before randomization and 2 and 4 weeks after randomization, but SF-36 is measured at randomization and 4 weeks after randomization.

**Discussion:**

The result of this trial (which will be available in 2012) will confirm whether acupuncture is effective to treat functional constipation and whether traditional acupuncture theories play an important role in it.

**Trials registration:**

Clinical Trials.gov NCT01411501

## Background

Functional constipation is a common disease, both in adults and in children. The prevalence in children ranges from 0.7% to 29.6%. In adults, functional constipation affects between 0.7% and 79% of the general population [1]. The prevalence is higher in females, older individuals, those of lower socioeconomic status, and those with a lower educational level [2,3]. As a common and well-recognized public health problem, functional constipation influences patient quality of life and consumes many healthcare resources [4,5].

Several therapies (such as lifestyle changes, osmotic agents, bulking agents, and so on) [6,7] have been used in clinical practice, which is believed to be helpful in relieving symptoms. Nonetheless, many of these therapies were not proven to be effective for the condition, or were difficult to tolerate because of adverse events [8-10].

Because of the unclear etiology and pathogenesis, the therapeutic options are relatively limited. As a result, complementary or alternative therapies, such as acupuncture, are attractive to both patients and practitioners. China has a long history of managing gastrointestinal symptoms through acupuncture, including diarrhea, constipation and gastroenteritis [11]. Results of several randomized controlled trials (RCTs) showed that acupuncture may be an effective treatment for functional constipation, by improving frequency and time of defecation, and the patient’s quality of life [12-14]. However, convincing evidence for the effectiveness of treating functional constipation with acupuncture is still inadequate, due to the poor quality of current studies. Therefore, we designed a multicenter randomized controlled trial to address these problems and hopefully provide a more conclusive answer to the questions.

In this trial, we first aim at investigating whether acupuncture is effective in treating functional constipation through a comparison with Mosapride Citrate control. Second, we will attempt to determine whether different acupuncture points are the same in managing this condition. The work reported in this article is financed by the National Basic Research Program (973 Program) in China, and is registered with an identifier (NCT01411501) in Clinical Trials.gov.

## Methods and design

### Design

**Figure 1:**
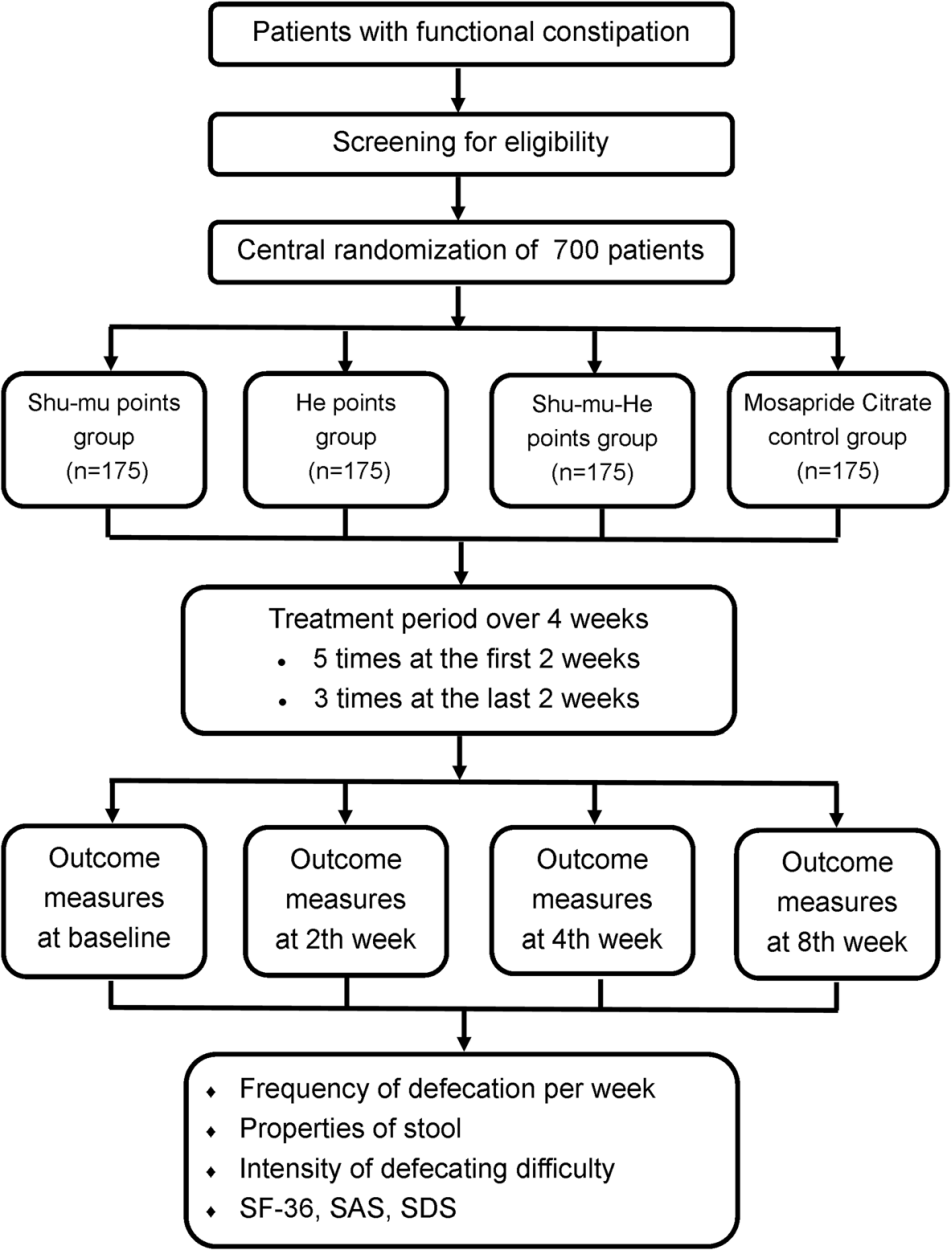
Trial flow chart.

**Figure 2:**
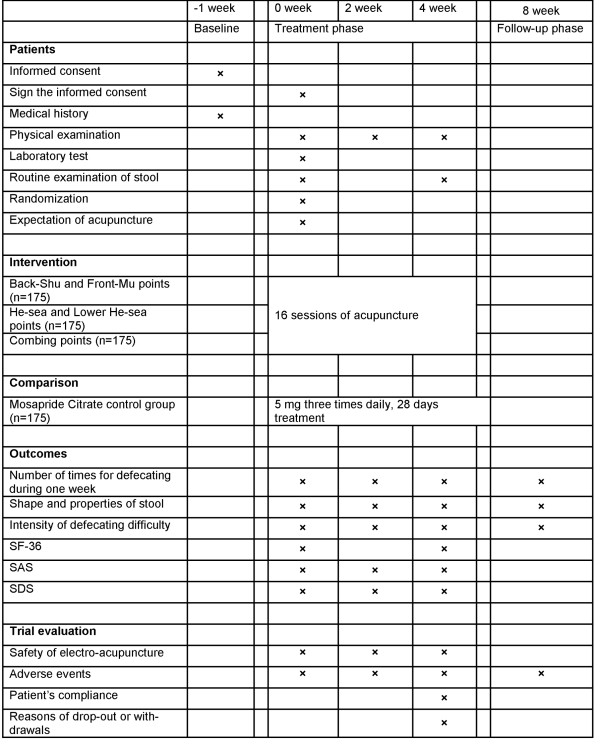
**Trial processes chart.** SF-36, MOS item Short Form health survey; SAS, Self-Rating Anxiety Scale; SDS, Self-rating Depression Scale.

This is a randomized controlled, four-arm, large-scale trial comparing three acupuncture groups (using different acupuncture points) with Mosapride Citrate control group (Figures 1 and 2). Seven hundred participants will be included from the following eight hospitals: Guang’anMen Hospital of China Academy of Chinese Medical Sciences; First Affiliated Hospital of Chengdu University of Traditional Chinese Medicine (TCM); Affiliated Hospital of Anhui College of TCM; Affiliated Hospital of Changchun University of TCM; Affiliated Hospital of Guangzhou University of TCM; Affiliated Hospital of Hunan University of TCM; Affiliated Hospital of Shandong University of TCM; and Affiliated Hospital of Shanxi College of TCM. These participants will be randomly assigned to four groups (three acupuncture groups and Mosapride Citrate control group) through central randomization in a 1:1:1:1 ratio. The central randomization system will be used and performed by the Clinical Evaluation Center at the China Academy of Chinese Medical Sciences in Beijing. A random number and group assignment will be immediately received by telephone, mobile phone, or website sent from the Clinical Evaluation Center.

Participants will receive 16 sessions of acupuncture treatment over 4 weeks, with a treatment frequency of five times a week in the first 2 weeks and three times a week in the last 2 weeks. Each session will last 30 min. The follow-up period lasts 4 weeks (5 to 8 weeks after randomization). Participants will be asked to fill in constipation diaries from 1 week before randomization to 9 weeks after randomization. All outcomes are assessed at baseline and 2, 4, and 8 weeks after randomization according to the diaries.

The total research period within this study is 9 weeks for each patient. All patients are asked to record constipation diaries for 1 week before randomization (baseline phase). If they are eligible for this trial, they will be asked to finish diaries from 0 to 8 weeks after randomization.

This trial protocol has been approved by local institutional review boards and ethics committees (April 2011), and follows the principles of the Declaration of Helsinki (Version Edinburgh 2000). Before randomization all patients will be requested to sign the written informed consent, meanwhile they will be given enough time to decide whether they are willing to participate this trial, or they choose other treatment options.

### Patients

#### Study population

This study focuses on patients with functional constipation, containing a sample size of 700 participants. There are many potential factors leading to constipation, such as food, drugs, and so on. To ensure the precision of results of this trial, we developed the following eligible criteria.

#### Inclusion criteria

Participants will be included if they fulfilled the following conditions: diagnosed with functional constipation according to the criteria of the Rome III [15]; aged between 18 and 75 years old; no use of gastrointestinal drugs 1 week before randomization; provided a signed written consent form.

#### Exclusion criteria

Patients with any of the following conditions will be excluded: constipation caused by other disorders (for example, irritable bowel syndrome, post-surgery constipation, and so on); psychosis (since acupuncture is an invasive procedure and needs a treatment duration of 30 min, to ensure safety of patients with psychosis, we do not plan to include them); pregnant women or women in lactation (it is still unclear whether acupuncture will lead to abortion or other side effects); bleeding disorders (the acupuncture procedure involves penetration of the skin, therefore we will exclude participants with thrombocytopenia, and so on); serious illnesses of the heart, liver, or kidney, or other severe diseases (there may be risks that acupuncture induces stress action and thus harms patients with serious illness).

#### Recruitment procedures

Two strategies will be used to recruit participants with functional constipation. The first is to recruit participants in outpatient clinics from the eight hospitals. The chief physicians of gastroenterology and colorectal department in each hospital were invited to attend a discussion of how to recruit patients. Research assistants will be sent to their departments to help screen participants. Moreover, posters of this trial were shown outside the clinics to attract possible candidates. Second, we will post advertisements through TV broadcasts, newspapers, and so on. In these advertisements, we will briefly introduce the population we want to include, and mention the free acupuncture treatments for participants who are eligible.

### Interventions and comparison

#### Rationale for acupuncture protocol

The acupuncture protocol in this trial was formed in two steps: first, we screen for a range of acupuncture points and stimulation methods through a systematic review of records of ancient books and acupuncture textbooks, as well as results of published articles; second, meetings were held to further screen for a standardized acupuncture protocol and reach consensus among acupuncture experts in China. Based on the theory of acupuncture and TCM, Back-Shu and Front-Mu points (short for Shu-Mu points) are the classic combination for the treatment of internal organ disease, which is located in the abdomen or lower back of the body, so Back-Shu and Front-Mu acupuncture points of Large Intestine meridians are supposed to be the best points prescription to treat Large Intestine disease. Additionally, He-Sea and Lower He-Sea acupuncture points of Large Intestine meridians are also positive points prescription, which is situated in the upper or lower limbs of the body, as stated: ’He points are the best points to treat internal organ’s disease’ in *Nei Jing*. In our hypothesis, the combination of Shu-Mu points and He-sea points will achieve better therapeutic outcomes, and all the acupuncture groups will be non-inferior to Mosapride Citrate. Therefore, groups in our trial are defined as: (1) Back-Shu and Front-Mu acupoints of Large Intestine meridians (Shu-Mu points) group; (2) He-Sea and Lower He-Sea acupoints of Large Intestine meridians (He points) group; (3) Combining used Back-Shu, Front-Mu, He-Sea, and Lower He-Sea acupoints of Large Intestine meridians (Shu-Mu-He points) group; and (4) Mosapride Citrate control group (Table 1).

**Table 1:** Details of acupuncture protocol

**Group names**	**Acupuncture points**	**De qi sensation**	**Stimulation**
Back-Shu and Front-Mu acupoints of Large Intestine meridians (Shu-Mu points group)	Tianshu (ST25) Dachangshu (BL25)	Yes	Both points are punctured bilaterally. Tianshu (ST25) is punctured perpendicularly 1–1.5 cun. Dachangshu (BL25) is punctured perpendicularly 0.8-1.2 cun
He-Sea and Lower He-Sea acupoints of Large Intestine meridians (He points group)	Quchi (LI11) Shangjuxu (ST37)	Yes	Both points are punctured bilaterally. Quchi (LI11) is punctured perpendicularly 0.5-1 cun. Shangjuxu (ST37) is punctured perpendicularly 1–2 cun
Combining used Back-Shu, Front-Mu, He-Sea, and Lower He-Sea acupoints of Large Intestine meridians(Shu-Mu-He points group)	Tianshu (ST25) Dachangshu (BL25) Quchi (LI11) Shangjuxu (ST37)	Yes	Stimulation is the same as above mentioned

### Acupuncture groups

All acupuncture points are punctured by filiform needles. After being needled, the points will be punctured again using auxiliary needles 2 mm lateral to the first needle, and to a depth of 2 mm without manual stimulation. G6805-1A electro-acupuncture apparatus (Shanghai Huayi, made in Jiangsu, China) is used for electric stimulation at every acupuncture point. Each acupuncture needle and auxiliary needle of each point is connected with electricity by G6805-1A for 30 min, using continuous wave. The stimulation frequency is 20 Hz. The stimulation intensity varies from 0.1 mA to 1.0 mA until the patients feel comfortable.

Sterile, disposable Hwato needles are used in this trial, which are made in Suzhou, China; they are 25 to 50 mm in length and 0.3 mm in diameter. The auxiliary needles are of the same brand with 13 mm in length and 0.16 mm in diameter. De qi sensation will be achieved in the acupuncture groups, through lifting and thrusting combined with twirling and rotating the needles. After retaining the needles for 30 min, all needles are taken out with clean cotton balls to avoid bleeding.

Every patient will receive 16 treatments in total over a period of 4 weeks. Five times a week, with once a day for 5 days continuously with a 2-day interval in the first 2 weeks; three times a week, every 2 days with a 1-day interval in the last 2 weeks.

### Control group

Participants will be orally given Mosapride Citrate tablet with a dose of 5 mg, three times a day for 4 weeks continuously.

### Outcome measurement

The primary outcome in this trial is the number of times of defecation throughout the fourth week after randomization. The primary outcome will be measured at the other time points (2 and 8 weeks after randomization) but as a secondary analysis. The second outcomes include the following five items: shape and properties of stool; intensity of defecating difficulty; MOS item Short Form health survey (SF-36); Self-Rating Anxiety Scale (SAS); and Self-rating Depression Scale (SDS). The first two of second outcomes are measured in 1 week before randomization and 2, 4, and 8 weeks after randomization. Other second outcomes are measured 1 week before randomization and 2 and 4 weeks after randomization, but SF-36 is measured before randomization and 4 weeks after randomization.

All patients have to fill in constipation diaries 1 week before randomization (baseline phase) and 2, 4, and 8 weeks after randomization.

All participants will receive routine tests of blood, urine, and stool, and electrocardiogram (ECG), liver function (ALT, AST), kidney function (BUN, Scr) before randomization, in order to exclude patients who have serious heart, liver, and kidney disease, or other severe disease. Only routine tests of stool should be done twice before randomization and after accomplishment of acupuncture treatment.

During the trial, all adverse events, including bleeding, hematoma, fainting, serious pain, local infection, and so on, are to be recorded during treatment and the follow-up period. Serious adverse events should be reported to the principal investigator immediately. All details will be documented and treatment options for rescue should be given at once. To guarantee the quality of the study, all acupuncturists are required to attend special training classes. The purpose of these training classes is for acupuncturists to understand all the details of this trial. The special training classes have theoretical and practical lessons. For instance, they will be trained to use the central randomization method, to fill in the case report form, to find the correct points, to manipulate the needles, to choose electro-acupuncture apparatus, and so on. After attending all training classes and passing the special training test, acupuncturists are qualified to perform this trial. Additionally, to ensure the quality of this trial, the clinical monitors will check the processes of the trial and documenting the details of the processes once a month in every hospital. Moreover, monitors named by the principle investigator will check the accuracy and validity of the original data from the clinical centers.

Patient drop-outs and their reasons, and patients’ compliance will be recorded at the fourth week after randomization.

### Sample size calculation and statistical analysis

According to the previous literature [16], an improvement of 2.6 times (standard deviation 2.4) for defecating during 1 week with functional constipation by prucalopride is reported, and a difference of 1.6 times between groups is considered to be of clinical relevance [16]. In this study, we anticipated an improvement of 4.0 times by Shu-Mu points group, 3.8 times by He points group, 5.2 times by Shu-Mu-He points group, and 2.6 times by Mosapride Citrate tablet group. A standard deviation of 6.0 is considered, to allow a relatively high heterogeneous variance in this population. Moreover, we mainly focus on the comparison of Shu-Mu-He points group *vs*. Mosapride Citrate tablet group. So the between-group comparison will be done as: first, Shu-Mu-He points group will be compared with Mosapride Citrate tablet group as the primary comparison, since the prescription of Shu-Mu-He points is considered to be better than the other two acupuncture protocols; second, comparisons among acupuncture groups will be performed also as exploratory analysis. So, we used GPower 3.1.3 [Franz Faul, Universität Kiel, Germany], and input the four group mean defecation rates as 4.0, 3.8, 5.2, and 2.6 per week, with a common assumed standard deviation of 6.0, giving a calculated effect size of 0.15. With four groups of equal size (175/group, 700 in total) this gives the study 90% power at a 5% level of significance to detect such an overall effect size using a one-way ANOVA fixed effects omnibus test, and assuming a 15% loss to follow-up. For the pairwise comparison of primary interest (Shu-Mu-He *vs*. mosapride citrate) the study has over 90% power at a 1% level of significance (to make some adjustment for multiple comparisons) to detect a mean difference of 2.6 (5.2 to 2.6) assuming the common standard deviation of 6.0 defecations with 175 participants per group.

Statistical analysis will be performed by the Clinical Evaluation Center of China Academy of Chinese Medical Sciences in Beijing. The statistician is blinded from the allocation of groups. SPSS13.0 and SAS9.0 statistical software packages will be used to analyze the data.

Prior to all analyses, a detailed statistical analysis protocol was developed. The intention-to-treat (ITT) population was defined as the patients who are randomized and received at least one treatment session. The per-protocol (PP) population was defined as the patients who completed the study and do not have major protocol violations. All analyses were based on the ITT population and PP population. And the result of the ITT analysis will be compared with that of the PP analysis to check whether the results are consistent.

First of all, baseline characteristics will be analyzed by descriptive statistics for each group. Then analysis is done to make comparisons among the treatment groups and control group. If a significant difference is found by comparison of the treatment groups and the control group, the next step is to make comparisons among three acupuncture points treatment groups in effectiveness. Multiple comparisons will be adjusted according to Bonferroni correction method.

The repeated measures analysis will be used in the different time points assessment. The Kruskal-Wallis test will be employed in the analysis of skewed distribution data. Analysis of variance (ANOVA) is used for numerical variables, and Chi-square test for categorical variables.

There is one acupuncturist in each center to do all three interventions, which probably introduces clustering effect in this trial. Therefore, we calculated the intracluster correlation coefficient from the results of this trial and report the coefficient [17].

## Discussions

The result of this trial is expected to provide convincing evidence that acupuncture is effective for patients with functional constipation. The trial is sponsored and financially supported by ’973 Program’ from the Ministry of Science and Technology of China, which is the most important basic research program based on clinical practice. Functional constipation is one of the 43 common diseases which the World Health Organization recommended acupuncture treatment for. However, currently there is not enough evidence for acupuncture based on strict clinical trial of evidence-based medicine (EBM) because of the poor quality of current studies, for example, small sample size, no description of methods for randomization, no standardized acupuncture protocol which may lead to performance bias, and so on. In this trial, we used central randomization, multicenter design, standardized acupuncture protocol, large sample size to ensure power, and so on. Therefore we conduct a multicenter randomized controlled large-scale trial to clarify the efficacy of acupuncture for treating functional constipation.

A major issue in planning the design of this trial is whether sham acupuncture should be used. Since the primary consideration of this trial is to clarify whether acupuncture is effective or whether acupuncture helps relieving symptoms of patients with functional constipation, we all agree that a pragmatic design is more suitable, which requires a positive drug as the control group. There is some evidence that Mosapride is effective for improvement of lower tract mobility, and thus is effective for management of constipation [18-20]. However, there is no proven drug clearly ahead of the rest. We choose Mosapride as a positive drug control based on: (1) it was reported to be superior to placebo for irritable bowel syndrome (IBS) patients with constipation, and it may have the potential to treat IBS patients with constipation and/or functional constipation [21]; and (2) a randomized controlled trial in China showed the same result [22], which indicates Mosapride may be a better option than other medication for Chinese population with functional constipation. So we choose Mosapride Citrate as a positive drug control. In this trial, we did not include a placebo acupuncture control, because we did not know whether acupuncture is effective for patients with functional constipation. Thus, if acupuncture is effective, then the next step would be to compare acupuncture with sham acupuncture to clarify absolute acupuncture treatment effect.

There are three different acupuncture groups in this trial, which are all expected to be effective according to traditional acupuncture theories. Combined use of Back-Shu and Front-Mu (Shu-Mu points) are regarded as the most commonly acupuncture prescription. In addition, He-Sea and Lower He-Sea points of Large Intestine meridians are also commonly used acupuncture prescription. The prescription of Shu-Mu-He points is composed of both Shu-Mu points (Shu-Mu points group, located at abdomen and lower-back) and He points (He points group, located at upper and lower limbs), which is expected to be the best therapeutic effect. Comparison of the three acupuncture groups will solve the question: whether combined use of Shu-Mu-He points will lead to better clinical outcome than using Shu-Mu or He points alone, which may give insights to whether traditional acupuncture theories work.

In this trial, we use 3:1 randomization design comparing three different types of acupuncture to Mosapride Citrate, but not to a sham acupuncture group or a placebo group. Because this trial is to answer whether acupuncture is effective and safe, regarding Mosapride Citrate is an effective drug for functional constipation, we compare acupuncture with Mosapride Citrate (positive drug control). Furthermore, although all three acupuncture points are believed to be effective for the condition, the combination of Shu-Mu-He points (Shu-Mu-He points group) is better than the other two (Shu-Mu points group and He points group), so the other two groups could be considered to be control groups (more powerful sham acupuncture groups than non-acupoints control groups) for Shu-Mu-He points group.

Before this trial, we ran a small sample randomized controlled trial of acupuncture for patients with functional constipation, with a follow-up period of 6 months [23]. The results showed that acupuncture at Tianshu (ST25) would increase the frequency of defecation after acupuncture treatment and last for about 1 month, so we choose to follow up participants for 4 weeks after treatment. Moreover, to get more information of acupuncture effect on functional constipation, we set an assessment period at 2 weeks after inclusion.

We focus on a specific population though this trial is a pragmatic design. First, we exclude patients with constipation caused by other disorders, especially for constipation caused by surgery and irritable bowel syndrome. Because constipation after surgery may be caused by anesthesia, pain medication, and so on, which acupuncture may not be helpful in these conditions according to the previous investigation [18,23]. Second, we exclude functional constipation patients accompanying other serious conditions, for example, heart failure, kidney failure, and so on, to ensure acupuncture would not do harm to these patients.

According to recommendations of a responder in clinical trials for functional gastrointestinal disorders [24], self-reported diaries will be used in this trial. The participants will know whether they receive acupuncture or medication, but will not know which type of acupuncture they have received, therefore, participants in Mosapride group might bias the outcome assessment. The primary outcome and the secondary outcomes will be calculated from the diaries. Considering that the frequency of defecation is the most important concern of patients with functional constipation, and that acupuncture may be of best effect at the end of the treatment [25,26], we choose the number of times for defecating during 1 week in the fourth week after randomization [24].

Due to lack of clear evidence of effectiveness with acupuncture to treating functional constipation, our trial aims to clarify this issue by means of a multicenter randomized controlled large-scale trial.

In conclusion, the results of this trial are expected to confirm whether acupuncture is effective in the management of functional constipation and whether traditional acupuncture theories play an important role in it.

## Trial status

The first participants were included on August 17, 2011, and this article was submitted on November 1, 2011. To date, 340 participants have been recruited.

## Competing interests

The authors declare that they have no competing interests.

## Authors’ contributions

YL, HZ, FZ, S Z, FZ, HbZ, MC, XhJ, YyC, BhJ, BZ, and ZsL participated in the conception and design of the trial, in plans for the analysis of the data, and in drafting the manuscript. FZ, FZ, HbZ, MC, XhJ, YyC, and BhJ participated in data collection, and were in charge of recruitment and treatment of patients in each center. All the authors discussed, read, revised the manuscript, and all approved the publication of this protocol.
